# TWAS-GKF: a novel method for causal gene identification in transcriptome-wide association studies with knockoff inference

**DOI:** 10.1093/bioinformatics/btae502

**Published:** 2024-08-27

**Authors:** Anqi Wang, Peixin Tian, Yan Dora Zhang

**Affiliations:** Department of Statistics and Actuarial Science, The University of Hong Kong, Hong Kong SAR, 999077, China; Department of Statistics and Actuarial Science, The University of Hong Kong, Hong Kong SAR, 999077, China; Department of Statistics and Actuarial Science, The University of Hong Kong, Hong Kong SAR, 999077, China

## Abstract

**Motivation:**

Transcriptome-wide association study (TWAS) aims to identify trait-associated genes regulated by significant variants to explore the underlying biological mechanisms at a tissue-specific level. Despite the advancement of current TWAS methods to cover diverse traits, traditional approaches still face two main challenges: (i) the lack of methods that can guarantee finite-sample false discovery rate (FDR) control in identifying trait-associated genes; and (ii) the requirement for individual-level data, which is often inaccessible.

**Results:**

To address this challenge, we propose a powerful knockoff inference method termed TWAS-GKF to identify candidate trait-associated genes with a guaranteed finite-sample FDR control. TWAS-GKF introduces the main idea of Ghostknockoff inference to generate knockoff variables using only summary statistics instead of individual-level data. In extensive studies, we demonstrate that TWAS-GKF successfully controls the finite-sample FDR under a pre-specified FDR level across all settings. We further apply TWAS-GKF to identify genes in brain cerebellum tissue from the Genotype-Tissue Expression (GTEx) v8 project associated with schizophrenia (SCZ) from the Psychiatric Genomics Consortium (PGC), and genes in liver tissue related to low-density lipoprotein cholesterol (LDL-C) from the UK Biobank, respectively. The results reveal that the majority of the identified genes are validated by Open Targets Validation Platform.

**Availability and implementation:**

The R package TWAS.GKF is publicly available at https://github.com/AnqiWang2021/TWAS.GKF.

## 1 Introduction

Genome-wide association study (GWAS) has identified thousands of trait-associated variants (Fine *et al.* 2019, Li *et al.* 2019). There are many methods to locate the candidate significant genes. However, many single nucleotide polymorphisms (SNPs) are located in noncoding regions, which complicates the interpretation of their biological functions and the translation of these associations into complex traits of scientific interest (Zhu *et al.* 2016, Khunsriraksakul *et al.* 2022, Shang *et al.* 2023). Despite these variants in noncoding regions having limited direct influence on protein-coding sequences, they can still induce epigenetic changes by regulating gene expression (Shang *et al.* 2023). Expression Quantitative Trait Loci (eQTL) data has emerged as a common strategy to overcome these challenges. Recent advancements in transcriptome-wide association study (TWAS) integrates eQTL data with GWAS results to elucidate associations between genes and complex traits. Specifically, TWAS applies eQTL data with an expression imputation model to identify trait-associated genes regulated by variants, even if they are far from each other. The inherent advantages of TWAS lie in its ability to provide valuable biological insights into the genetic basis of complex traits.

The fundamental workflow of TWAS can be structured into three sequential steps. First, TWAS estimates the effect between multiple genetic variants and gene expression by fitting a multivariate regression model, employing a small reference panel, such as the Genotype-Tissue Expression (GTEx) v8 project data (Aguet *et al.*, 2020) and the Encyclopedia of DNA Elements (ENCODE) project (Mai *et al.* 2023). For example, PrediXcan utilizes individual-level data to fit a marginal linear regression model of multiple cis-SNPs on gene expression using an elastic net model (Gamazon *et al.* 2015). Second, TWAS applies some fitted predictive models to impute gene expression data of GWAS individuals. Third, hypothesis tests are conducted between the imputed expression levels and the trait to identify candidate genes. In general, for individual-level data, existing methods utilize regression models (e.g. linear, or logistic) to estimate effect sizes and corresponding *P*-values. Subsequently, these methods implement Bonferroni correction (Dunn 1961) and Benjamini-Hochberg correction (Benjamini and Hochberg 1995) to adjust *P*-values and select candidate genes whose *P*-values are under a pre-specified significant level. However, due to the limited accessibility of individual-level data, methods such as S-PrediXcan (Barbeira *et al.* 2018) and FUSION (Gusev *et al.* 2016) have been proposed to identify trait-associated genes using regulatory weights and GWAS summary statistics without imputing gene expression. Specifically, S-PrediXcan is the summary statistics version of PrediXcan, using only GWAS summary statistics to estimate *Z*-scores (Pividori *et al.* 2023). FUSION utilizes Bayesian sparse linear mixed models (BSLMM) to estimate the effect sizes of SNPs on gene expression. However, both the elastic net model and the BSLMM model have limitations in capturing the distribution of complex genetic architecture (Nagpal *et al.* 2019). Nonetheless, conventional association tests used in previous TWAS methods cannot provide a guarantee of finite-sample false discovery rate (FDR) control.

To address this challenge, we propose a novel method named TWAS-GKF to identify candidate causal genes by integrating the concept of Ghostknockoff with a guaranteed finite-sample FDR control (He *et al.* 2022). Knockoff inference was initially proposed by Barber and Candès (2015) to control finite-sample FDR. The key idea is to construct “mirror” variables, termed knockoff variables, which have a similar structure to the original covariates but are not associated with the response variable. The original variables whose correlations with the response variable are significantly stronger than their knockoff variables are selected as candidate variables. Specifically, vanilla knockoffs (model-X knockoffs), which utilize individual-level data to construct synthetic knockoff variables serving as negative controls for feature selection to achieve finite-sample FDR control (Candès *et al.* 2018). Due to the difficulty in accessing individual-level data, He *et al.* (2022) proposed Ghostknockoff, which uses *Z*-scores from summary statistics instead of individual-level data. Ghostknockoff enhances the identification of causal SNPs by reducing the confounding effects of linkage disequilibrium (LD) blocks. Moreover, Ma *et al.* (2023) presented a novel gene test, BIGKnock, which incorporates knockoff inference to handle large-scale biobank data. BIGKnock leverages long-range chromatin interaction data and yields results which are independent of potentially biased training data.

Our proposed method, TWAS-GKF, is a powerful knockoff inference approach that brings the idea of Ghostknockoff to generate multiple knockoff variables using *Z*-scores from summary statistics, which allows for variable selection in the second stage of TWAS, addressing the limitations of conventional association tests. Due to Monte Carlo sampling, the results of vanilla knockoffs are unstable and difficult to reproduce (Gimenez and Zou 2019, Nguyen *et al.* 2020). In response to this, TWAS-GKF borrows the innovative concept of multiple knockoffs (Gimenez and Zou 2019), which generates multiple knockoff variables simultaneously to facilitate stability. Specifically, TWAS-GKF has three main steps. First, we estimate *Z*-scores of candidate genes and compute the correlation matrix among them from the LD matrix by using the relationship between genes and SNPs. Second, we generate Ghostknockoff variables according to the correlation matrix of the genes and the estimated *Z*-scores. Based on this, we compute two knockoff statistics to leverage the relevance of the candidate genes. To measure the effect of the genes on the trait of scientific interest, we further introduce a novel feature statistic, named GReX feature statistic (GFS). Third, we determine a data-dependent threshold with a pre-specified FDR level based on the knockoff statistics and select the genes whose GFSs exceed the data-dependent threshold.

We evaluate the performance of our proposed method, TWAS-GKF, through empirical simulations and real applications. Our empirical findings reveal that TWAS-GKF can always effectively control finite-sample FDR with better stability across all examined cases. In real applications, we apply the TWAS-GKF and S-PrediXcan methods to brain cerebellum and liver tissues from the Genotype-Tissue Expression (GTEx) v8 project (Aguet *et al.*, 2020) , respectively, to identify genes associated with schizophrenia (SCZ) from the Psychiatric Genomics Consortium (PGC, Trubetskoy *et al.* (2022)) and low-density lipoprotein cholesterol (LDL-C) from the UK Biobank (Sudlow *et al.* 2015). We compare the results of TWAS-GKF with those of S-PrediXcan. Notably, TWAS-GKF exhibit a significant improvement in the power of variable selection. Specifically, TWAS-GKF identifies 135 genes associated with SCZ in brain cerebellum tissue and 130 genes related to LDL-C in liver tissue, respectively. In contrast, S-PrediXcan identifies 106 genes for SCZ and 91 genes for LDL-C, separately. Importantly, most of the genes identified by S-PrediXcan are encompassed within the set identified by TWAS-GKF, highlighting the high power and accuracy of our proposed method.

## 2 Materials and methods

### 2.1 Data overview

In this study, we request the GWAS summary statistics for schizophrenia (SCZ) from Psychiatric Genomics Consortium (PGC), which includes 74 776 cases and 101 023 controls (Trubetskoy *et al.* 2022). In their GWAS study, Trubetskoy *et al.* (2022) conducted an association meta-analysis to obtain the summary statistics by analyzing up to 7 585 078 variants from 9 cohorts with a minor allele frequency (MAF) greater than or equal to 1%. On the other hand, the summary statistics for low-density lipoprotein cholesterol (LDL-C) are obtained from UK Biobank, which recruited 500 000 participants aged 37–73 years from 2006 to 2010 (Sudlow *et al.* 2015). In addition, the variance of each gene is estimated by utilizing the linkage disequilibrium (LD) matrix calculated with the R package “bigsnpr,” based on 3717 nearly independent LD blocks partitioned by the method described by Li *et al.* (2024) using the 1000 Genome reference panel (Auton *et al.* 2015). Gene expression data for brain cerebellum tissue and the liver tissue are sourced from GTEx v8, which analyzes 15 201 RNA sequencing (RNA-seq) across 49 tissue from 838 postmortem donors (Aguet *et al.*, 2020). In total, the brain cerebellum and liver tissues consist of 7274 and 4706 genes, respectively.

### 2.2 Overview of S-PrediXcan

Summary-PrediXcan (S-PrediXcan), proposed by Barbeira *et al.* (2018), is a summary-level approach that calculates the association between predicted gene expression and complex traits using GWAS summary statistics. In order to impute gene expression, for gene *g*, the relationship between the gene expression level (GReX) $X_{g}$ and the genotype matrix $G_{g}$ can be modeled as follows (Gamazon *et al.* 2015):

(1) $$\begin{array}{cc} X_{g}= & \alpha_{X}+\sum_{j}w_{j,g}G_{j,g}+\varepsilon_{X}\\ & =\alpha_{X}+W_{g}G_{g}+\varepsilon_{X},\;g\in [D],j\in [J], \end{array}$$

where [D] denotes the set of {1,2,…,D}, [J] denotes the set of $\{1,2,\ldots ,J\},\;G_{j,g}$ is the *j*th column of genotype matrix $G_{g}$, representing the number of reference alleles for single-nucleotide polymorphism (SNP) *j* (e.g. 0, 1, 2), and $W_{g}$ is a vector of $w_{j,g}$, representing the effect sizes of the $G_{g}$ on gene *g*. Particularly, S-PrediXcan estimates the gene expression by ${\hat{X}}_{g}=\sum_{j}{\hat{w}}_{j,g}G_{j,g}$ for each gene *g*, where ${\hat{w}}_{j,g}$ is the estimate of the effect size $w_{j,g}$ in Equation (1) for each gene using an elastic net model (Zou and Hastie 2005).

To test the association between gene *g* and the complex trait, S-PrediXcan models the target trait ***Y*** using the following regression models with the predicted gene expression ${\hat{X}}_{g}$ and $G_{j,g}$, respectively (Barbeira *et al.* 2018):

(2) $$Y=\alpha_{Y_{1}}+\sum_{g}{\hat{X}}_{g}\beta_{g}+\varepsilon_{Y_{1}},$$

and

(3) $$Y=\alpha_{Y_{2}}+G_{j,g}\gamma_{j,g}+\varepsilon_{Y_{2}},$$

where $Y=\left(Y_{1},Y_{2},\ldots ,Y_{n}\right)$ is an *n*-dimensional vector representing the phenotype for *n* independent individuals, $\alpha_{Y_{1}}$ and $\alpha_{Y_{2}}$ are intercepts, *β_g_* represents the effect size of predicted gene expression of gene *g* on the trait, $\gamma_{j,g}$ is the effect size for SNP *j* of gene *g*, and $\varepsilon_{Y_{1}}$ and $\varepsilon_{Y_{2}}$ are error terms independent of ${\hat{X}}_{g}$ and $G_{j,g}$, respectively. Define $\hat{\mathrm{Cov}}$ and $\hat{\mathrm{Var}}$ as the operators to calculate the sample covariance and variance, respectively. Let ${\hat{\sigma }}_{g}^{2}=\hat{\mathrm{Var}}({\hat{X}}_{g})=W_{g}^{\prime }{\hat{R}}_{g}W_{g}$ represent the variance of predicted gene expression of gene *g*, where ${\hat{R}}_{g}=\hat{\mathrm{Var}}\left(G_{g}\right)$ denotes the LD matrix of the SNPs of gene *g*. By combining Equations (1) and (2), the estimated effect size ${\hat{\beta }}_{g}$ and its corresponding standard error $\mathrm{se}({\hat{\beta }}_{g})$ can be derived as (Barbeira *et al.* 2018):

(4) $${\hat{\beta }}_{g}=\frac{\hat{\mathrm{Cov}}\left({\hat{X}}_{g},Y\right)}{\hat{\mathrm{Var}}\left({\hat{X}}_{g}\right)}=\frac{\sum_{j}w_{j,g}{\hat{\gamma }}_{j,g}{\hat{\rho }}_{j}^{2}}{{\hat{\sigma }}_{g}^{2}},$$

and

(5) $${\mathrm{se}}^{2}({\hat{\beta }}_{g})=\frac{{\hat{\sigma }}_{Y}^{2}-{\hat{\beta }}_{g}^{2}{\hat{\sigma }}_{g}^{2}}{n{\hat{\sigma }}_{g}^{2}},$$

where

$${\hat{\gamma }}_{j,g}=\frac{\hat{\mathrm{Cov}}\left(G_{j,g},Y\right)}{\hat{\mathrm{Var}}\left(G_{j,g}\right)}=\frac{\hat{\mathrm{Cov}}\left(G_{j,g},Y\right)}{{\hat{\rho }}_{j}^{2}},$$

${\hat{\rho }}_{j}^{2}=\hat{\mathrm{Var}}\left(G_{j,g}\right)$ is the estimated variance of SNP *j* in gene *g*, corresponding to the *j*th diagonal element of ${\hat{R}}_{g},\;{\hat{\sigma }}_{Y}^{2}$ is the sample variance of the trait, and ${\hat{\gamma }}_{j,g}$ is the estimate of the *j*th SNP effect size for gene *g*.

S-PrediXcan exclusively utilizes summary statistics of GWAS data to estimate the effect sizes of genes using the Equation (4) and their corresponding *P*-values. The Bonferroni Correction (Dunn 1961) and Benjamini-Hochberg correction (Benjamini and Hochberg 1995) are employed to adjust these *P*-values and select candidate genes whose adjusted *P*-values are smaller than a pre-specified significance level. This approach enables the identification of genes that are likely to exhibit a significant association with the trait of interest, relying solely on summary-level data from GWAS. However, S-PrediXcan cannot provide finite-sample FDR control. Moreover, S-PrediXcan relies on *P*-values to select candidate genes, which may require either a reasonable assumption of data distribution or a large number of replications to obtain valid *P*-values for controlling FDR (Ge *et al.* 2021).

### 2.3 Gene selection with finite-sample FDR control using TWAS-GKF

We propose a novel *P*-value-free framework, TWAS-GKF, to control finite-sample FDR and improve power using freely available GWAS data. We construct GhostKnockoff knockoffs which only require *Z*-scores of the candidate genes from summary statistics and do not need strong assumptions on the distribution of *Z*-scores. The goal of TWAS-GKF is to identify the genes with nonzero effect sizes. Specifically, our proposed method includes three main steps.

First, the *Z*-score for gene *g* can be obtained by using the Equations (4) and (5) to calculate the ratio of estimated effect size ${\hat{\beta }}_{g}$ and its standard error $\mathrm{se}({\hat{\beta }}_{g})$ as follows:

(6) $$Z_{g}=\frac{{\hat{\beta }}_{g}}{\mathrm{se}\left({\hat{\beta }}_{g}\right)}\approx \sum_{j}w_{j,g}\frac{{\hat{\rho }}_{j}}{{\hat{\sigma }}_{g}}\frac{{\hat{\gamma }}_{j,g}}{\mathrm{se}\left({\hat{\gamma }}_{j,g}\right)},$$

where

$${\mathrm{se}}^{2}\left({\hat{\gamma }}_{j,g}\right)=\frac{{\hat{\sigma }}_{Y}^{2}-{\hat{\gamma }}_{j,g}^{2}{\hat{\rho }}_{j}^{2}}{n{\hat{\rho }}_{j}^{2}}.$$

Second, we incorporate the idea of GhostKnockoff (He *et al.* 2022) to select candidate genes associated with the trait of scientific interest with finite-sample FDR control. Suppose the correlation matrix of candidate genes is denoted as a *D *×* D* matrix Σ (the expression of Σ is detailed in the Supplementary Materials). We generate *M* Ghostknockoff variables ${\left\{{\tilde{Z}}^{m}\right\}}_{m=1}^{M}$ of the *Z*-scores for all candidate genes simultaneously following:

(7) $${\tilde{Z}}^{m}=\Theta Z+\Lambda ,$$

where ${\tilde{Z}}^{m}$ is a *D*-length vector of *m*th Ghostknockoff variable of *Z*-scores, $Z=\left(Z_{1},\ldots ,Z_{D}\right)$ denotes a *D*-dimensional vector of *Z*-scores for *D* genes, Λ is from a normal distribution N(0,V) with variance $V=2\Phi -\Phi \Sigma^{-1}\Phi ,\;\Theta$ is represented as $\left(I-\Phi \Sigma^{-1}\right)$, and I is a *D *×* D* identity matrix. We obtain $\Phi =\mathrm{diag}\left(s_{1},\ldots ,s_{D}\right)$ by solving the following convex optimization problem:

$$\;\text{minimize}\;\sum_{g}\left|1-s_{g}\right|,\;\text{subject to}\;\{\begin{array}{c} \frac{M+1}{M}\Sigma -\Phi ≽0,\\ s_{g}\geq 0,g\in [D], \end{array}$$

where the first constraint requires $\frac{M+1}{M}\Sigma -\Phi$ to be positive semidefinite. In this way, we show in the Supplementary Materials that our Ghostknockoff variables ${\tilde{Z}}^{m}$ constructed in Equation (7) retain the two key properties of knockoff inference (Candès *et al.* 2018, He *et al.* 2022):

1. For any subset S⊆[D] and $m\in [M],\;{\left(Z,{\tilde{Z}}^{m}\right)}_{\text{swap}(S)}\overset{d}{=}(Z,{\tilde{Z}}^{m})$, where ${\left(Z,{\tilde{Z}}^{m}\right)}_{\text{swap}(S)}$ is obtained by swapping the *k*th element of ***Z*** and ${\tilde{Z}}^{m}$ for each k∈S;
2.
${\tilde{Z}}^{m}⊥Y|Z$ for any m∈[M].

To leverage the relevance of the candidate genes, we compute the intermediate feature statistics $\left(T_{g},{\tilde{T}}_{g}^{m}\right)$ for gene *g*, which can also be considered as important scores, with corresponding mathematical expressions:

$$T_{g}=Z_{g}^{2},\;{\tilde{T}}_{g}^{m}={\left({\tilde{Z}}_{g}^{m}\right)}^{2}.$$

Based on the intermediate feature statistics, we further compute the knockoff statistics, whose expressions are represented as:

(8) $$\kappa_{g}=\max(T_{g},{\tilde{T}}_{g}^{m}),\tau_{g}=T_{g}-\underset{m}{\mathrm{median}}{\tilde{T}}_{g}^{\left(m\right)},m\in [M],$$

where ${\tilde{T}}_{g}^{m}$ represents the intermediate feature statistics of the *m*th Ghostknockoff variable, ${\tilde{T}}_{g}^{\left(m\right)}$ represents the intermediate feature statistics of the *m*th Ghostknockoff variable ordered in ascending order, and *κ_g_* is the Ghostknockoff variable that has the largest important scores among the original variables and Ghostknockoff variables, and *τ_g_* measures the difference between the largest intermediate feature statistics and their median value, which can be treated as the magnitude of invariance for swapping. If *M *=* *1, the problem would be about single knockoff procedure, which is equivalent to the model-X knockoff filter (Candès *et al.* 2018). Otherwise, the problem will be about multiple knockoff inference with application of knockoff statistics *κ_g_* to measure whether the important score of gene *g* is larger than that of its Ghostknockoff variable. Moreover, we introduce a novel feature statistic, GReX feature statistic (GFS), $\Delta =(\delta_{1},\delta_{2}\ldots ,\delta_{D})$, whose *g*th entry is:

(9) $$\delta_{g}=\left(T_{g}-\underset{m}{\mathrm{median}}{\tilde{T}}_{g}^{m}\right)I_{T_{g}\geq \max{\tilde{T}}_{g}^{m}},m\in [M]$$

where Δ measure the effects from genes to the trait.

Third, we compute a data-dependent threshold to control finite-sample FDR:

(10) $$\tau =\min\left\{t>0:\frac{\frac{1}{M}+\frac{1}{M}\#\left\{\kappa_{g}\geq 1,\tau_{g}\geq t\right\}}{\#\left\{\kappa_{g}=0,\tau_{g}\geq t\right\}}\leq \alpha \right\},$$

where *α* is a pre-specified FDR level. We finally select the candidate genes whose GFSs satisfy the following requirement:

$$\hat{S}=\{g\in [D]:\delta_{g}>\tau \}.$$

The data-dependent threshold established in Equation (10) allows for a controlled estimate of the FDR to guarantee the property of knockoff inference. TWAS-GKF incorporates the idea of GhostKnockoffs to select candidate genes with finite-sample FDR control instead of conducting hypothesis test. Specifically, TWAS-GKF constructs Ghostknockoff variables corresponding to the *Z*-scores of candidate genes obtained from the GWAS summary statistics instead of generating individual-level knockoff variables, offering computational efficiency while still preserving the key properties of knockoff inference. TWAS-GKF is free of assumptions on the distribution of *P*-values compared with S-PrediXcan. Moreover, TWAS-GKF takes gene-gene correlation into account, which improves the performance of TWAS-GKF in candidate genes identification. In this article, we evaluate the performance using the modified false discovery rate (mFDR) (Candès *et al.* 2018) and true positive proportion (TPP):

$$\text{mFDR}=E[\frac{\#\{g\in \hat{S}\cap H_{0}\}}{\#\hat{S}+1/\alpha }],\;\text{TPP}=E[\frac{\#\{g\in \hat{S}\cap H_{1}\}}{\#H_{1}}],$$

where $H_{0}=\{g:\beta_{g}=0\}$ includes the candidate genes with no association with the target trait, and $H_{1}=\{g:\beta_{g}\neq 0\}$ includes the genes associated with the target trait. The implementation details are available in Algorithm 1.

Algorithm 1. Algorithm of TWAS-GKF

**Input:**

 ${\left\{W_{g}\right\}}_{g=1}^{D}$ - a list of gene-specific SNP weights for *D* genes;

 ${\left\{{\hat{\gamma }}_{g}\right\}}_{g=1}^{D}$ - a list of genetic effect estimates from GWAS summary statistics;

 ${\left\{\mathrm{se}({\hat{\gamma }}_{g})\right\}}_{g=1}^{D}$ - a list of standard errors of genetic effect estimates from GWAS summary statistics;

 ${\left\{{\hat{R}}_{g}\right\}}_{g=1}^{D}$ - a list containing LD matrics for *D* genes;

 Σ - a *D *×* D* correlation matrix of genes;

 *α* - a pre-specified FDR level;

 *M–*number of multiple knockoff variables.

**Output:** Variable selection set: $\hat{S}$.

1: Calculate *Z*-scores for the genes using Equation (6);

2: Generate *M* knockoff variables ${\left\{{\tilde{Z}}_{g}^{m}\right\}}_{m=1}^{M}$ of the *Z*-scores using Equation (7);

3: Compute the knockoff statistics based on Equation (8);

4: Apply Equation (9) to obtain GREx feature statistics (GFSs): $\Delta =(\delta_{1},\delta_{2}\ldots ,\delta_{D})$;

5: Variable selection set: $\hat{S}=\left\{g\in [D]:\delta_{g}>\tau \right\}$, where *τ* is a data-dependent threshold defined in Equation (10).

## 3 Simulation studies

We conduct a wide range of simulation settings to assess the performance of our proposed method and S-PrediXcan. To mimic the real data, we extract the genotypes from UK Biobank data, which undergo a quality control procedure following Chang *et al.* (2015). Specifically, we exclude the missing samples, and select the SNPs whose *P*-values of Hardy–Weinberg equilibrium Fisher’s exact test are smaller than $1\times 10^{-5}$ with MAF greater than 1%. Considering computational efficiency, we focus on SNPs located on chromosomes 1 and 19 because these two chromosomes have the largest number of recorded genes in brain cerebellum tissue from GTEx v8 (Aguet *et al.*, 2020) with a total of *D *=* *863 genes.

**Figure 1.**
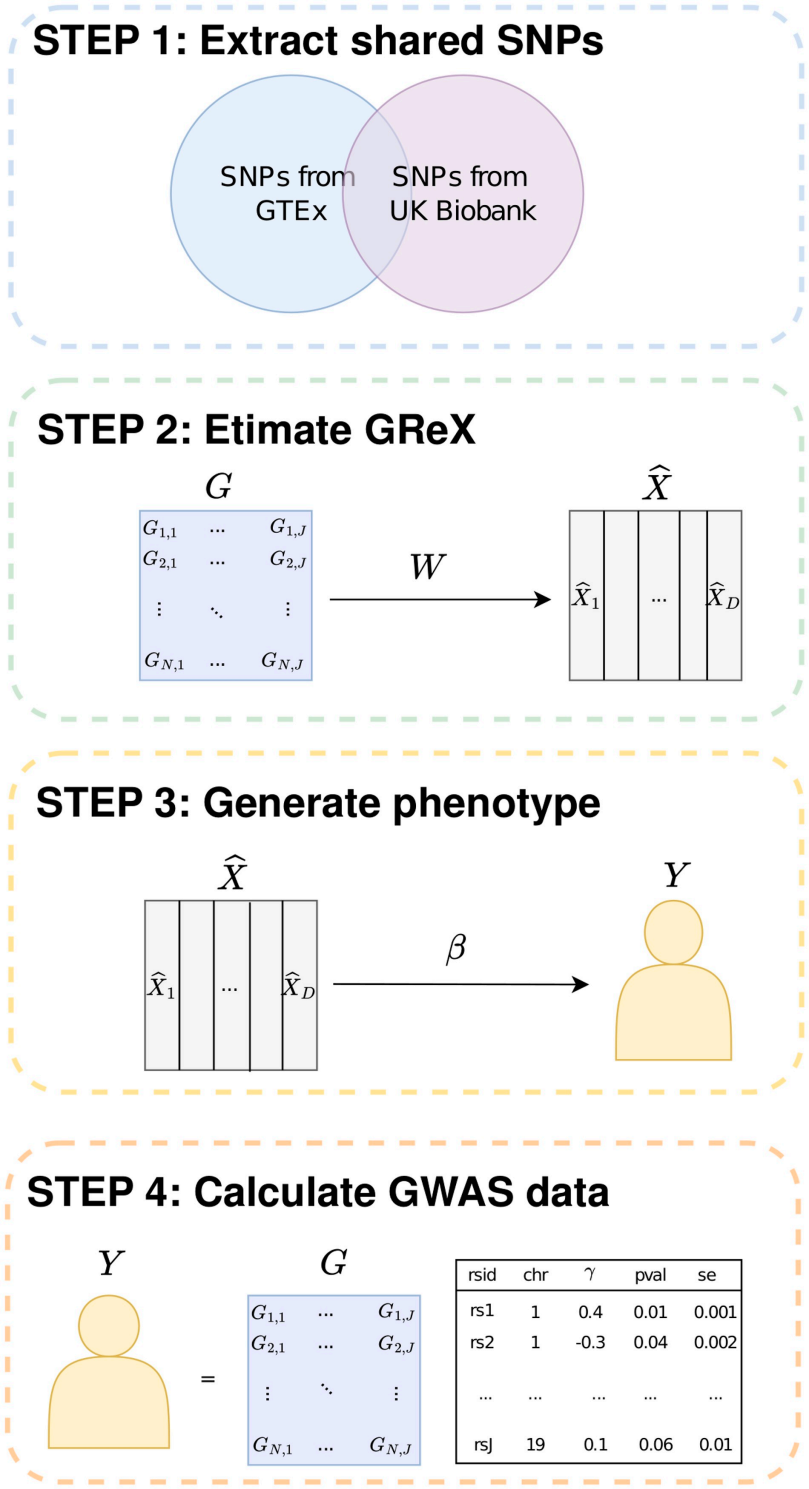
Flowchart of data generation process. First, we extract the shared SNPs between GTEx v8 data and UK Biobank data. Second, we estimate gene expression levels based on the SNPs. Third, we generate the phenotype vector based on the predicted gene expression. Fourth, we compute the GWAS summary statistics and *Z*-scores.

Our data generation process contains four steps, as depicted in Fig. 1. First, we extract the 90 308 SNPs which are located in the 863 candidate genes and shared by UK Biobank and GTEx v8 data. Following the estimation step of PrediXcan (Gamazon *et al.* 2015), we obtain the estimated effect sizes $\left\{{\hat{\omega }}_{j,g}\right\}$ of SNPs on the candidate genes by fitting the marginal elastic net model. Second, we compute the gene expression level ${\hat{X}}_{g}$. Third, we randomly assign causal genes with nonzero effect according to the causal ratio *η* among 2%, 5%, and 10%. Then we simulate the phenotype vector ***Y*** as follows:

$$Y=\sum_{g=1}^{D}{\hat{X}}_{g}\beta_{g}+\epsilon ,$$

where ${\hat{X}}_{g}$ is the predicted gene expression level. The effect sizes ${\left\{\beta_{g}\right\}}_{g=1}^{D}$ are generated as follows: if gene *g* is not a causal gene, then $\beta_{g}=0$; otherwise $\beta_{g}∼N\left(0,\frac{h^{2}}{k}\right)$, where $h^{2}\in \{0.05,0.1,0.2\}$ is the heritability representing the overall variance explained by our simulated genes, k=⌈η×D⌉ is the number of causal genes, and ⌈x⌉ represents the smallest integer no less than the real number *x*. The error terms ϵ are independently generated from $N\left(0,1-h^{2}\right)$. Fourth, we calculate the GWAS summary statistics by fitting marginal regression models of simulated phenotypes on each standardized SNP. Furthermore, we calculate *Z*-scores with the estimated effect sizes according to Equations (4) and (6). We set the pre-specified mFDR level α=0.05 in Equation (10), the number of multiple knockoff variables *M *=* *5, and the sample size n∈{50 000,100 000,150 000}. For S-PrediXcan, we apply the Benjamini-Hochberg (BH) correction to adjust *P*-values and select genes whose adjusted *P*-values are smaller than the significance level $\alpha_{\mathrm{adj}}=0.05$.

**Figure 2.**
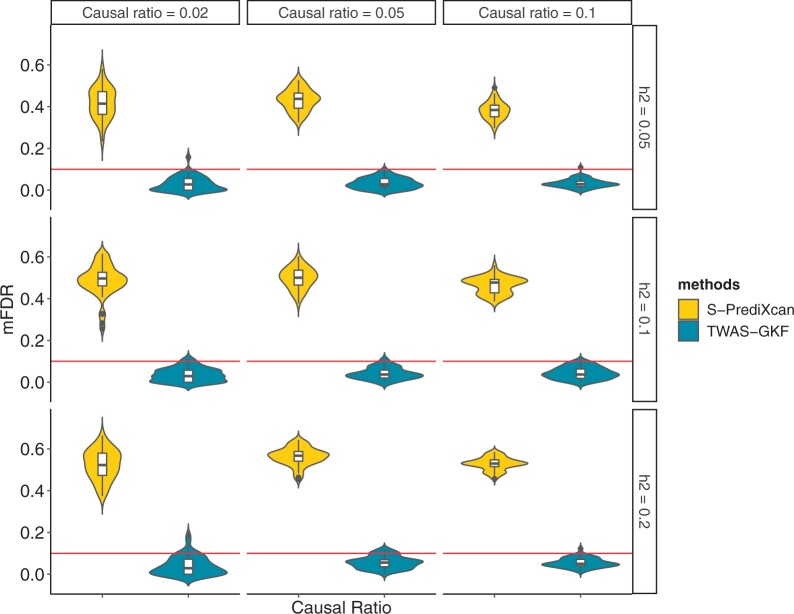
Violin plot and box plot illustrate the mFDR of TWAS-GKF and S-PrediXcan with the sample size of N=150 000, for different values of ratio (η=2%,5%,10%) and gene expression heritability ($h^{2}=0.05,0.1,0.2$), the mFDR results for our proposed method TWAS-GKF are displayed on the right side, while the mFDR values of the competing method S-PrediXcan is shown on the left side. The width of the plot corresponds to the density of the mFDR values, with the broader shape indicating the denser distribution of results.

We apply TPP and mFDR to evaluate the variable selection performance for both TWAS-GKF and S-PrediXcan. The average mFDR and TPP values can be found in Table 1, with a pre-specified mFDR level of 0.05. We also report the estimated standard errors in Supplementary Table S1. Notably, TWAS-GKF always controls mFDR under the pre-specified level of 0.05. However, even though we apply BH correction to adjust the *P*-values of S-PrediXcan, it still fails to control mFDR under 0.05. The average mFDR value for S-PrediXcan can even exceed 0.5 under settings where the sample size is 150 000 and *h*^2^ is 0.2. With an increase in sample size (*n*), both methods perform with higher mFDR and TPP values. TWAS-GKF still effectively controls mFDR under the specified level, albeit with slightly conservative power. In Fig. 2, we observe that the mFDR of TWAS-GKF performs more stable than the mFDR of S-PrediXcan, as the violin plot of S-PrediXcan is more widely distributed. Furthermore, the standard errors of mFDR for S-PrediXcan are higher than those for TWAS-GKF, indicating greater stability in variable selection for TWAS-GKF.

**Table 1.** The mean of mFDR and TPP of causal genes selection of TWAS-GKF and S-PrediXcan across 50 simulations with $\alpha =0.05,\;\alpha_{\text{adj}}=0.05$ and *M *=* *5.

*n*	*h* ^2^	Causal ratio	S-PrediXcan		TWAS-GKF	
			TPP	mFDR	TPP	mFDR
50 000	0.05	0.02	0.786	0.305	0.629	0.029
		0.05	0.710	0.317	0.546	0.028
		0.10	0.615	0.283	0.441	0.033
	0.10	0.02	0.861	0.358	0.719	0.048
		0.05	0.796	0.390	0.635	0.049
		0.10	0.730	0.361	0.551	0.043
	0.20	0.02	0.886	0.450	0.728	0.050
		0.05	0.849	0.461	0.703	0.056
		0.10	0.809	0.432	0.626	0.050
100 000	0.05	0.02	0.864	0.362	0.691	0.029
		0.05	0.802	0.391	0.605	0.023
		0.10	0.729	0.363	0.531	0.037
	0.10	0.02	0.900	0.438	0.756	0.042
		0.05	0.842	0.448	0.674	0.045
		0.10	0.808	0.422	0.612	0.045
	0.20	0.02	0.933	0.485	0.789	0.050
		0.05	0.905	0.515	0.735	0.044
		0.10	0.865	0.494	0.668	0.054
150 000	0.05	0.02	0.886	0.417	0.727	0.030
		0.05	0.841	0.432	0.656	0.033
		0.10	0.764	0.382	0.586	0.033
	0.10	0.02	0.926	0.486	0.737	0.036
		0.05	0.872	0.498	0.696	0.041
		0.10	0.834	0.464	0.640	0.044
	0.20	0.02	0.951	0.524	0.778	0.040
		0.05	0.918	0.563	0.740	0.056
		0.10	0.889	0.528	0.686	0.055

Given S-PrediXcan’s inflated mFDR, it is crucial to consider both mFDR and TPP when comparing the methods. Therefore, we adjust the significance level of BH correction to keep the mFDR performance of the two methods closely aligned (in Table 2, Supplementary Tables S2 and S3). The results demonstrate that when the mFDR performance of the two methods is close, TWAS-GKF exhibits higher power in variable selection. We further report the mFDR performance of S-PrediXcan under various significance levels in Supplementary Fig. S1. The results indicate that the mFDR performance of S-PrediXcan always exceeds 0.2, even when the significance level is $1\times 10^{-10}$. This demonstrates that S-PrediXcan exhibits poor performance in the accuracy of variable selection.

**Table 2.** The mean of mFDR and TPP of causal genes selection of TWAS-GKF and S-PrediXcan across 50 simulations with $\alpha =0.2,\;\alpha_{\text{adj}}=1\times 10^{-10}$ and *M *=* *5.

*n*	*h* ^2^	Causal ratio	S-PrediXcan		TWAS-GKF	
			TPP	mFDR	TPP	mFDR
150 000	0.05	0.02	0.752	0.209	0.787	0.186
		0.05	0.612	0.211	0.728	0.177
		0.10	0.455	0.163	0.662	0.170

In summary, the simulation studies demonstrate that TWAS-GKF consistently achieves finite-sample mFDR control across all settings, whereas S-PrediXcan shows higher TPP values but with inflated mFDR. S-PrediXcan is more likely to select more genes, resulting in higher power but subject to false-positive inflation. Specifically, when the mFDR performance of these two methods is comparable, TWAS-GKF performs more powerful in variable selection compared to S-PrediXcan.

## 4 Real data analysis

In this section, we apply the TWAS-GKF and S-PrediXcan methods to brain cerebellum and liver tissues from the GTEx v8 project (Aguet *et al.*, 2020) to identify genes related to two common traits, SCZ from the PGC (Trubetskoy *et al.* 2022) and LDL-C from the UK Biobank (Sudlow *et al.* 2015). To calculate the *Z*-score for each candidate gene in these two tissues according to Equation (5), we first use the elastic net model to estimate the SNP effect sizes $\left\{{\hat{\omega }}_{j,g}\right\}$ for gene *g*. We then extract the estimated effect sizes $\left\{{\hat{\omega }}_{j,g}\right\}$, along with GWAS summary statistics and the corresponding LD matrix for SNPs within gene *g*, which intersect with SCZ summary statistics and LD reference panel dataset from the 1000 Genomes project (Auton *et al.* 2015). The LD matrix is obtained using the method proposed by Li *et al.* (2024). To ensure the consistent directionality of effect sizes across the GWAS summary statistics and SNP effect sizes $\left\{{\hat{\omega }}_{j,g}\right\}$ for all candidate genes, we check for the allele flipping. Finally, we calculate a correlation matrix across candidate genes using gene expressions directly obtained from the GTEx v8 project for each tissue. For S-PrediXcan method, we apply BH correction to adjust *P*-values and genes with adjusted *P*-values lower than the significant level $\alpha_{\mathrm{adj}}=0.05$ are considered as potential causal genes. For our proposed method, we set the number of multiple knockoff variables at *M *=* *5 and follow Algorithm 1 to select genes maintaining a pre-specified FDR level of α=0.05.

**Figure 3.**
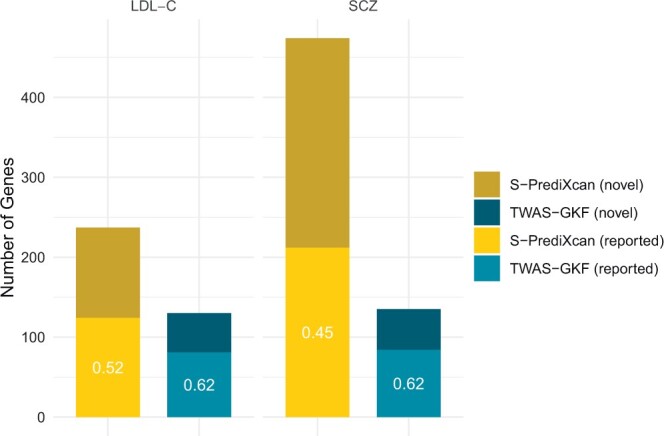
The ratios of genes identified by TWAS-GKF and S-PrediXcan that are validated by Open Targets Validation Platform. “S-PrediXcan (reported)” and “TWAS-GKF (reported)” refer to genes identified by these two methods that overlap with platform. “S-PrediXcan (novel)” and “TWAS-GKF (novel)” demonstrate genes identified by these two methods that are not intersected with the platform. The ratios of identified genes that are validated are reported in the figure.

The analysis of the brain cerebellum tissue dataset, consisting of 7274 genes, reveals that S-PrediXcan identifies 474 candidate genes for SCZ. On the other hand, our proposed method, TWAS-GKF, identifies a total of 135 candidate genes. We then compare our findings with the Open Targets Validation Platform (https://platform.opentargets.org/), a data integration and visualization platform developed by Koscielny *et al.* (2017) that serves as the “gold standard” for providing evidence on the association between known and potential drug targets and diseases. The results show that 84 out of the 135 candidate genes identified by our proposed method are validated by this platform, as detailed in Supplementary Table S4. In comparison, 212 out of the 474 candidate genes from S-PrediXcan are validated. Although S-PrediXcan identifies more genes than our proposed method, the ratio of genes validated by platform to those identified by S-PrediXcan (45%) is much lower than that of TWAS-GKF (62%), as illustrated in Fig. 3. This suggests that many candidate genes identified by S-PrediXcan may not be causal, consistent with simulation studies that demonstrate that S-PrediXcan fails to control FDR. It’s noteworthy that some extra novel significant genes identified by TWAS-GKF are also reported by existing literature. For example, Chen *et al.* (2019) detected a novel heterozygous frameshift deletion in the CHST9 gene, associated with SCZ in the Chinese population. This deletion found in three children diagnosed with schizophrenia across two separate families was confirmed via PCR-based Sanger sequencing. Athanasiu *et al.* (2010) conducted a GWAS study of SCZ using a Norwegian discovery sample and found that the marker rs11662668 located in the RBBP8 gene is associated with SCZ, establishing the association between RBBP8 and SCZ. Moreover, Stauffer *et al.* (2023) utilized Hi-C-coupled MAGMA (H-MAGMA) method in a GWAS study on SCZ and identified a significant association with PLEKHM1. This gene encodes a multivalent endocytic adaptor that regulates autophagy pathways through lysosome fusion events (McEwan *et al.* 2015), crucial mechanisms in SCZ pathophysiology (Merenlender-Wagner *et al.* 2015). In addition, EMC2 plays a key role in the ER-protein processing pathways, fundamental to the cellular pathophysiology of SCZ, by facilitating in the insertion and folding of protein into the ER (Kim *et al.*2019, O’Donnell *et al.* 2020).

The liver tissue comprises a total of 4706 genes. TWAS-GKF identifies 130 candidate genes, while S-PrediXcan identifies 237 genes. Among the 130 genes obtained by TWAS-GKF, 81 are found in the “gold standard” list (as shown in Supplementary Table S5) and 124 out of the 237 genes identified by S-PrediXcan are validated by the Open Targets Validation Platform. The ratio of genes found in the “gold standard” list to those detected by TWAS-GKF method (62%) is still higher than that of S-PrediXcan (52%), as shown in Fig. 3. Our proposed method, TWAS-GKF identifies several well-known genes associated with LDL-C which are not in the gold standard list. For example, Jiang *et al.* (2017) identified PROZ, as a potential new biomarker for pulmonary tuberculosis (TB). Since the blood coagulation and lipid indices, such as LDL-C, are associated with TB, PROZ may influence LDL-C levels, potentially contributing to TB. Moreover, Petrelis *et al.* (2022) found that the SNP rs6921438 is robustly associated with both HDL-C and LDL-C plasma levels. This intergenic SNP, located between LOC100132354 (lncRNA) and the C6orf223 gene, interacts with rs2375981 from the ZADH2 gene, indicating a link between ZADH2 and LDL-C levels. MAPRE2, which is commonly regulated by GPSM1, exhibits a positive correlation of expression with LDL-C (Yan *et al.* 2022). In addition, ACOT1, a critical cytosolic enzyme involved in fatty acid (FA) metabolism, regulates PPAR*α* which is associated with LDL-C level, indicating an association between ACOT1 and LDL-C (Franklin *et al.* 2017, Li *et al.* 2021, Ortega-Meléndez *et al.* 2022).

In summary, we applied our proposed method, TWAS-GKF, with the brain cerebellum and liver tissues from the GTEx v8 project to identify candidate genes associated with SCZ from the PGC and LDL-C from the UK Biobank, respectively. The results show that most of the genes identified by our proposed TWAS-GKF are reported as causal genes, consistent with the simulation studies demonstrating the accuracy of TWAS-GKF in gene selection.

## 5 Discussion

In this article, we propose a novel knockoff-based inference named TWAS-GKF for identifying causal genes with a guarantee of finite-sample false discovery rate (FDR) control. Our proposed method incorporates the main idea of Ghostknockoff inference to generate multiple knockoff variables using the *Z*-scores from GWAS summary statistics and further identify candidate genes while maintaining control of FDR. The empirical results demonstrate that our method can efficiently control FDR under the pre-specified FDR level across all settings compared to the competing method S-PrediXcan. Furthermore, we apply TWAS-GKF and S-PrediXcan methods identify the genes related with SCZ from PGC in brain cerebellum tissue and genes associated with LDL-C from UK Biobank in liver tissue, respectively. The results indicate that TWAS-GKF outperforms S-PrediXcan in gene identification, providing more stable FDR control.

TWAS-GKF offers several advantages. First, it only requires summary statistics for gene selection, which are more easily accessible compared to individual-level data, and it accounts for correlation across genes to enhance gene identification. Second, TWAS-GKF exhibits enhanced power in variable selection while ensuring finite-sample FDR control by. Third, our method introduces new knockoff statistics, GFSs, making it as the first approach to incorporate knockoff inference within TWAS methods.

Despite these advantages, there are also some limitations to our method. First, TWAS-GKF’s performance relies on the accuracy of predicted gene expressions. Second, we compute the variance of each gene based on the LD matrix, which would ideally be calculated using an in-sample LD matrix. Mismatching between the LD target sample and reference panel may result in higher FDR.

## Supplementary Material

btae502_Supplementary_Data

## Data Availability

The data that support the findings of this study can be downloaded from the following sources: all protected data of the GTEx project are available through the database of Genotypes and Phenotypes (dbGaP) (accession number phs000424.v8.p2) at https://www.ncbi.nlm.nih.gov/projects/gap/cgi-bin/study.cgi?study_id=phs000424.v8.p2, the GWAS data can be found as PGC GWAS summary statistics (https://pgc.unc.edu/), and UK Biobank summary statistics (https://broad-ukb-sumstats-us-east-1.s3.amazonaws.com/round2/additive-tsvs/30790_irnt.gwas.imputed_v3.both_sexes.varorder.tsv.bgz), the genotype data from the UK Biobank are available under accession number 140822.
