# Supplementary material for: TWAS-GKF: a novel method for causal gene identification in transcriptome-wide association studies with knockoff inference
Source: Bioinformatics. 2024 Aug 27;40(8):btae502. doi: 10.1093/bioinformatics/btae502 (PMC11361808; doi:10.1093/bioinformatics/btae502)
Supplement: btae502_Supplementary_Data [file btae502_supplementary_data.zip › TWAS_GKF_bioinformatics_supp.pdf]

Supplementary materials to 'TWAS-GKF: A Novel Method for Causal Gene Identification in Transcriptome-wide Association Studies with Knockoff Inference'

Anqi Wang[1†], Peixin Tian[1†], Yan Dora Zhang[1*]

[1]Department of Statistics and Actuarial Science, The University of Hong Kong, Hong Kong SAR, China.

July 2, 2024

S1. Methods

S1.1 Exchangeability

Proof. To prove that original variable $\boldsymbol{Z}$ and knockoff variables $\widetilde{\boldsymbol{Z}}^m$ satisfy the exchangeability condition, we need to show that the joint distribution of $\left(\boldsymbol{Z}, \widetilde{\boldsymbol{Z}}^m\right)$ remains invariant when any subset of original variables is swapped with their corresponding knockoff variables. Note that the Z-score of gene g, denoted as Z_g, can be rewritten as follows:

$$Z_g = \frac{\boldsymbol{W}_g^* \cdot \boldsymbol{Z}_{\text{SNPs},g}}{\boldsymbol{W}_g^{*\prime} \cdot \boldsymbol{\Sigma}_g \cdot \boldsymbol{W}_g^*}, \tag{A1}$$

where $\boldsymbol{W}_g^* = (\widehat{\rho}_1 w_{1,g}, \ldots, \widehat{\rho}_J w_{J,g})'$, and

$$\boldsymbol{Z}_{\text{SNPs},g} = (Z_{1,g}, \ldots, Z_{J,g})' = \left(\frac{\widehat{\gamma}_{1,g}}{\text{se}\left(\widehat{\gamma}_{1,g}\right)}, \ldots, \frac{\widehat{\gamma}_{J,g}}{\text{se}\left(\widehat{\gamma}_{J,g}\right)}\right)'$$

the correlation matrix $\boldsymbol{\Sigma}_g$ is defined as:

$$\boldsymbol{\Sigma}_g = \text{diag}\left(\frac{1}{\widehat{\rho}_1}, \ldots, \frac{1}{\widehat{\rho}_J}\right) \cdot \widehat{\text{Var}}\left(\boldsymbol{X}_g\right) \cdot \text{diag}\left(\frac{1}{\widehat{\rho}_1}, \ldots, \frac{1}{\widehat{\rho}_J}\right)$$

Thus, according to the equation (A1), the Z_g is normally distributed:

$$Z_g \sim \mathcal{N}\left(0, \frac{\boldsymbol{W}_g^* \cdot \boldsymbol{\Sigma}_{\text{SNPs},g}}{\sqrt{\boldsymbol{W}_g^{*\prime} \cdot \boldsymbol{\Sigma}_g \cdot \boldsymbol{W}_g^*}}\right),$$

†These authors contributed equally to this work.
*To whom correspondence should be addressed.

where $\boldsymbol{\Sigma}_{\text{SNPs},g}$ is the correlation matrix of SNPs contained in gene g. Then we can obtain the distribution of $\boldsymbol{Z} = (Z_1, \ldots, Z_D)'$ with the covariance matrix denoted as $\boldsymbol{\Sigma}$:

$$\boldsymbol{Z} \sim \mathcal{N}(\boldsymbol{0}, \boldsymbol{\Sigma}),$$

where the diagonal elements of $\boldsymbol{\Sigma}$ are the variance of each gene $\frac{\boldsymbol{W}_g^* \cdot \boldsymbol{\Sigma}_{\text{SNPs},g}}{\sqrt{\boldsymbol{W}_g^{*\prime} \cdot \boldsymbol{\Sigma}_g \cdot \boldsymbol{W}_g^*}}$, and nondiagonal elements are the covariance between different genes (Z_g and Z_h) which can be written as:

$$\text{Cov}(Z_g, Z_h) = \frac{\mathbb{E}\left[\left(\boldsymbol{W}_g^{*\prime} \cdot Z_{\text{SNPs},g}\right)\left(\boldsymbol{W}_h^{*\prime} \cdot Z_{\text{SNPs},h}\right)\right]}{\sqrt{\left(\boldsymbol{W}_g^{*\prime} \cdot \boldsymbol{\Sigma}_g \cdot \boldsymbol{W}_g^*\right)\left(\boldsymbol{W}_h^{*\prime} \cdot \boldsymbol{\Sigma}_h \cdot \boldsymbol{W}_h\right)}}.$$

Given the distribution of original variable $\boldsymbol{Z}$, we use equation (7) in Section 2.3 to generate the Ghostknockoff variables. Therefore, the conditional distribution of $\widetilde{\boldsymbol{Z}}^m$ given by the original variable $\boldsymbol{Z}$ can be expressed as:

$$\widetilde{\boldsymbol{Z}}^m \mid \boldsymbol{Z} \sim \mathcal{N}(\boldsymbol{\mu}, \boldsymbol{\Omega}),$$

where $\boldsymbol{\mu} = \boldsymbol{Z}\left(\boldsymbol{I} - \boldsymbol{\Sigma}^{-1}\boldsymbol{\Phi}\right)$ and $\boldsymbol{\Omega} = 2\boldsymbol{\Phi} - \boldsymbol{\Phi}\boldsymbol{\Sigma}^{-1}\boldsymbol{\Phi}$. Since

$$\mathbb{E}\left(\widetilde{\boldsymbol{Z}}^m\right) = \mathbb{E}\left(\mathbb{E}\left(\widetilde{\boldsymbol{Z}}^m \mid \boldsymbol{Z}\right)\right) = \mathbb{E}\left(\boldsymbol{Z}\left(\boldsymbol{I} - \boldsymbol{\Sigma}^{-1}\boldsymbol{\Phi}\right)\right) = \boldsymbol{0},$$

then the joint distribution of $\left(\boldsymbol{Z}, \widetilde{\boldsymbol{Z}}^m\right)$ is normally distributed with covariance matrix $\boldsymbol{\Gamma}$:

$$\left(\boldsymbol{Z}, \widetilde{\boldsymbol{Z}}^m\right) \sim \mathcal{N}(\boldsymbol{0}, \boldsymbol{\Gamma}).$$

To further construct the covariance matrix, we compute the $\text{Var}(\boldsymbol{Z})$, $\text{Var}\left(\widetilde{\boldsymbol{Z}}^m\right)$ and $\text{Cov}\left(\boldsymbol{Z}, \widetilde{\boldsymbol{Z}}^m\right)$. We can directly obtian the value of $\text{Var}(\boldsymbol{Z})$, which is equal to $\boldsymbol{\Sigma}$, and

$$\begin{aligned}
\text{Var}\left(\widetilde{\boldsymbol{Z}}^m\right) &= \mathbb{E}\left(\text{Var}\left(\widetilde{\boldsymbol{Z}}^m \mid \boldsymbol{Z}\right)\right) + \text{Var}\left(\mathbb{E}\left(\widetilde{\boldsymbol{Z}}^m \mid \boldsymbol{Z}\right)\right) \\
&= \mathbb{E}\left(2\boldsymbol{\Phi} - \boldsymbol{\Phi}\boldsymbol{\Sigma}^{-1}\boldsymbol{\Phi}\right) + \text{Var}\left(\boldsymbol{Z}\left(\boldsymbol{I} - \boldsymbol{\Sigma}^{-1}\boldsymbol{\Phi}\right)\right) \\
&= 2\boldsymbol{\Phi} - \boldsymbol{\Phi}\boldsymbol{\Sigma}^{-1}\boldsymbol{\Phi} + \left(\boldsymbol{I} - \boldsymbol{\Sigma}^{-1}\boldsymbol{\Phi}\right)' \Sigma \left(\boldsymbol{I} - \boldsymbol{\Sigma}^{-1}\boldsymbol{\Phi}\right) \\
&= \boldsymbol{\Sigma}, \\
\text{Cov}\left(\boldsymbol{Z}, \widetilde{\boldsymbol{Z}}^m\right) &= \text{Cov}\left(\boldsymbol{Z}, \boldsymbol{Z}\left(\boldsymbol{I} - \boldsymbol{\Sigma}^{-1}\boldsymbol{\Phi}\right) + \boldsymbol{\Lambda}\right) \\
&= \boldsymbol{\Sigma}\left(\boldsymbol{I} - \boldsymbol{\Sigma}^{-1}\boldsymbol{\Phi}\right) + \text{Cov}(\boldsymbol{\Sigma}, \boldsymbol{\Lambda}) \\
&= \boldsymbol{\Sigma} - \boldsymbol{\Phi}.
\end{aligned}$$

Thus, the covariance matrix $\boldsymbol{\Gamma}$ is as follows (Candes et al., 2018):

$$\boldsymbol{\Gamma} = \begin{pmatrix} \boldsymbol{\Sigma} & \boldsymbol{\Sigma} - \boldsymbol{\Phi} \\ \boldsymbol{\Sigma} - \boldsymbol{\Phi} & \boldsymbol{\Sigma} \end{pmatrix}.$$

Let $\boldsymbol{P}$ be the permutation matrix encoding the swap. We finally need to establish that

$$\boldsymbol{P}\boldsymbol{\mu}\boldsymbol{P}' = \boldsymbol{\mu}, \qquad \boldsymbol{P}\boldsymbol{\Gamma}\boldsymbol{P}' = \boldsymbol{\Gamma}, \tag{A2}$$

where $\boldsymbol{P}\boldsymbol{\mu}\boldsymbol{P}'$ and $\boldsymbol{P}\boldsymbol{\Gamma}\boldsymbol{P}'$ are the mean and covariance matrix obtained by swapping original with their corresponding knockoff variables, respectively. The first equality in (A2) holds because $\boldsymbol{\mu} = \mathbf{0}$. Based on the property of permutation matrix, $\boldsymbol{P}\boldsymbol{\Gamma}\boldsymbol{P}' = \boldsymbol{\Gamma}$ also holds, thus the joint distribution keeps invariant.

We use a simple example to explain the second equality in (A2). Assume $D = 3$, $S = \{2, 3\}$, and

$$\boldsymbol{\Gamma} = \begin{pmatrix} \sigma_{11} & \sigma_{12} & \sigma_{13} & \sigma_{11} - s_1 & \sigma_{12} & \sigma_{13} \\ \sigma_{12} & \sigma_{22} & \sigma_{23} & \sigma_{12} & \sigma_{22} - s_2 & \sigma_{23} \\ \sigma_{13} & \sigma_{23} & \sigma_{33} & \sigma_{13} & \sigma & \sigma_{33} - s_3 \\ \sigma_{11} - s_1 & \sigma_{12} & \sigma_{13} & \sigma_{11} & \sigma_{12} & \sigma_{13} \\ \sigma_{12} & \sigma_{22} - s_2 & \sigma_{23} & \sigma_{12} & \sigma_{22} & \sigma_{23} \\ \sigma_{13} & \sigma_{23} & \sigma_{33} - s_3 & \sigma_{13} & \sigma_{23} & \sigma_{33} \end{pmatrix},$$

thus,

$$\begin{aligned} &\boldsymbol{P}\boldsymbol{\Gamma}\boldsymbol{P}' \\ =& \begin{pmatrix} 1 & 0 & 0 & 0 & 0 & 0 \\ 0 & 0 & 0 & 0 & 1 & 0 \\ 0 & 0 & 0 & 0 & 0 & 1 \\ 0 & 0 & 0 & 1 & 0 & 0 \\ 0 & 1 & 0 & 0 & 0 & 0 \\ 0 & 0 & 1 & 0 & 0 & 0 \end{pmatrix} \boldsymbol{\Gamma} \begin{pmatrix} 1 & 0 & 0 & 0 & 0 & 0 \\ 0 & 0 & 0 & 0 & 1 & 0 \\ 0 & 0 & 0 & 0 & 0 & 1 \\ 0 & 0 & 0 & 1 & 0 & 0 \\ 0 & 1 & 0 & 0 & 0 & 0 \\ 0 & 0 & 1 & 0 & 0 & 0 \end{pmatrix}' \\ =& \boldsymbol{\Gamma}. \end{aligned}$$

S2. Simulation

Table S1: The standard errors of mFDR and TPP of causal genes selection of TWAS-GKF and S-PrediXcan across 50 simulations with $\alpha = 0.05$, $\alpha_{\text{adj}} = 0.05$ and $M = 5$.

n	h2	ratio	S-PrediXcan		TWAS-GKF	
			TPP	mFDR	TPP	mFDR
50000	0.05	0.02	0.087	0.078	0.118	0.033
		0.05	0.070	0.061	0.077	0.022
		0.10	0.062	0.046	0.067	0.024
	0.10	0.02	0.080	0.088	0.113	0.043
		0.05	0.066	0.059	0.072	0.032
		0.10	0.057	0.038	0.059	0.026
	0.20	0.02	0.074	0.073	0.116	0.040
		0.05	0.049	0.057	0.058	0.030
		0.10	0.042	0.043	0.050	0.028
100000	0.05	0.02	0.091	0.083	0.109	0.032
		0.05	0.068	0.056	0.074	0.029
		0.10	0.052	0.038	0.062	0.022
	0.10	0.02	0.065	0.085	0.097	0.044
		0.05	0.051	0.054	0.081	0.034
		0.10	0.040	0.038	0.048	0.022
	0.20	0.02	0.061	0.087	0.114	0.039
		0.05	0.049	0.047	0.070	0.034
		0.10	0.032	0.035	0.050	0.026
150000	0.05	0.02	0.080	0.074	0.114	0.034
		0.05	0.059	0.051	0.072	0.026
		0.10	0.043	0.042	0.048	0.021
	0.10	0.02	0.066	0.076	0.102	0.032
		0.05	0.047	0.050	0.068	0.028
		0.10	0.046	0.040	0.056	0.028
	0.20	0.02	0.046	0.071	0.111	0.044
		0.05	0.041	0.042	0.076	0.030
		0.10	0.031	0.031	0.058	0.022

Table S2: The average values of mFDR and TPP of causal genes selection of TWAS-GKF and S-PrediXcan across 50 simulations with $\alpha = 0.05$, $\alpha_{\text{adj}} = 1 \times 10^{-10}$ and $M = 5$.

n	h2	ratio	S-PrediXcan		TWAS-GKF	
			TPP	mFDR	TPP	mFDR
50000	0.05	0.02	0.546	0.134	0.711	0.184
		0.05	0.373	0.104	0.618	0.154
		0.10	0.212	0.086	0.535	0.171
	0.10	0.02	0.698	0.165	0.782	0.188
		0.05	0.513	0.176	0.703	0.179
		0.10	0.381	0.139	0.639	0.185
	0.20	0.02	0.744	0.241	0.817	0.184
		0.05	0.655	0.227	0.757	0.172
		0.10	0.533	0.204	0.706	0.176
100000	0.05	0.02	0.689	0.169	0.169	0.170
		0.05	0.535	0.178	0.178	0.170
		0.10	0.375	0.149	0.149	0.182
	0.10	0.02	0.793	0.240	0.240	0.194
		0.05	0.634	0.216	0.216	0.182
		0.10	0.529	0.213	0.213	0.177
	0.20	0.02	0.838	0.301	0.301	0.214
		0.05	0.749	0.279	0.279	0.176
		0.10	0.646	0.269	0.269	0.171
150000	0.05	0.02	0.752	0.209	0.787	0.186
		0.05	0.612	0.211	0.728	0.177
		0.10	0.455	0.163	0.662	0.170
	0.10	0.02	0.807	0.287	0.808	0.190
		0.05	0.719	0.273	0.767	0.182
		0.10	0.601	0.238	0.717	0.182
	0.20	0.02	0.867	0.331	0.864	0.194
		0.05	0.794	0.350	0.820	0.192
		0.10	0.706	0.310	0.759	0.187

Table S3: The standard errors of mFDR and TPP of causal genes selection of TWAS-GKF and S-PrediXcan across 50 simulations with $\alpha = 0.05$, $\alpha_{\text{adj}} = 1 \times 10^{-10}$ and $M = 5$.

n	h2	ratio	SPrediXcan		TWAS-GKF	
			TPP	mFDR	TPP	mFDR
50000	0.05	0.02	0.113	0.074	0.074	0.074
		0.05	0.072	0.039	0.039	0.039
		0.10	0.044	0.052	0.052	0.052
	0.10	0.02	0.112	0.073	0.073	0.073
		0.05	0.072	0.054	0.054	0.054
		0.10	0.046	0.045	0.045	0.045
	0.20	0.02	0.090	0.077	0.077	0.077
		0.05	0.068	0.053	0.053	0.053
		0.10	0.050	0.043	0.043	0.043
100000	0.05	0.02	0.124	0.069	0.096	0.093
		0.05	0.076	0.049	0.060	0.061
		0.10	0.054	0.042	0.061	0.058
	0.10	0.02	0.069	0.076	0.072	0.089
		0.05	0.085	0.068	0.065	0.072
		0.10	0.050	0.044	0.041	0.059
	0.20	0.02	0.095	0.088	0.086	0.086
		0.05	0.072	0.055	0.054	0.073
		0.10	0.042	0.051	0.049	0.041
150000	0.05	0.02	0.101	0.074	0.102	0.092
		0.05	0.083	0.053	0.069	0.066
		0.10	0.053	0.046	0.050	0.050
	0.10	0.02	0.094	0.078	0.090	0.088
		0.05	0.060	0.050	0.055	0.060
		0.10	0.049	0.047	0.054	0.053
	0.20	0.02	0.075	0.086	0.076	0.093
		0.05	0.062	0.045	0.059	0.060
		0.10	0.043	0.042	0.054	0.041

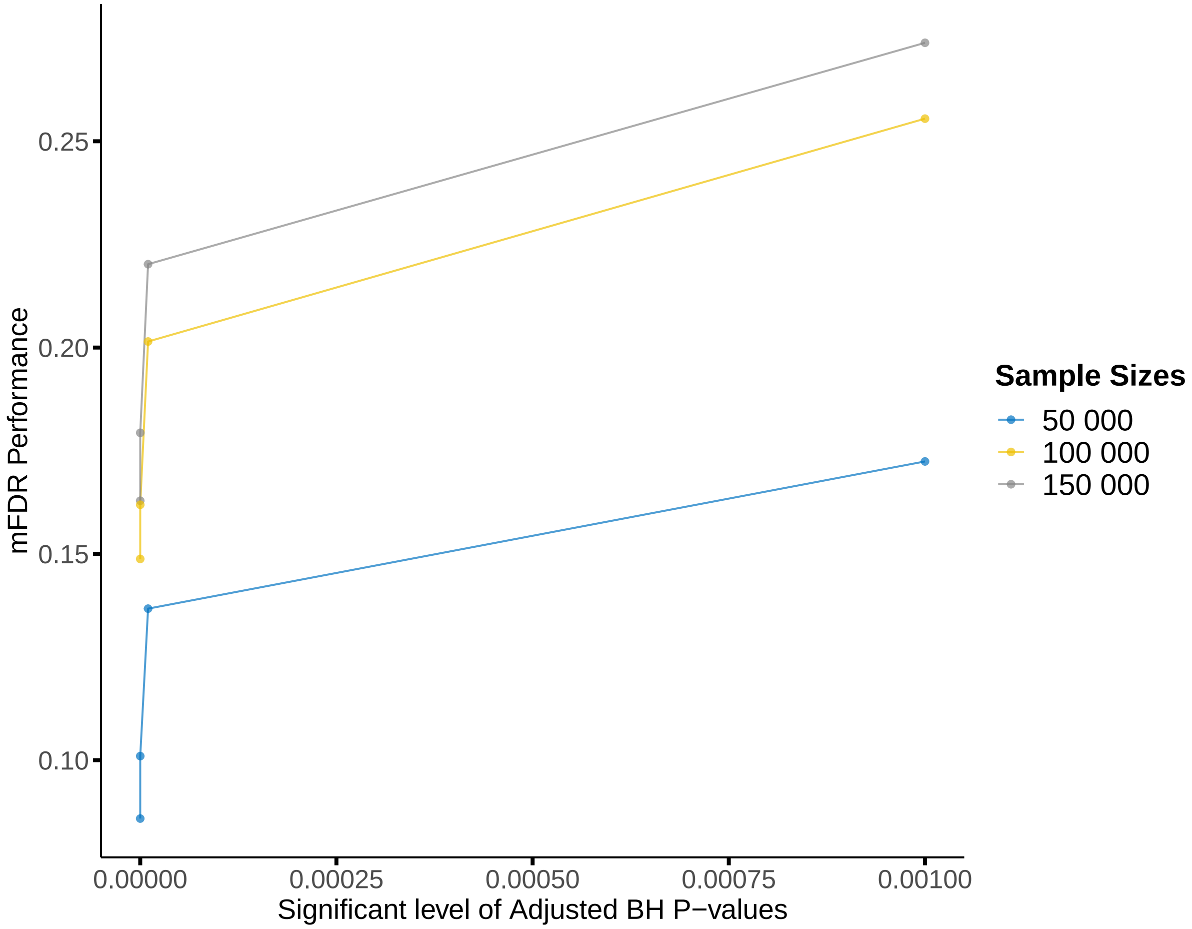

Figure S1: The mFDR performance of S-PrediXcan across 50 replications.

S3. Real Data Analysis

Table S4: The 84 candidate gene lists for SCZ validated by the Open Targets Validation Platform.

Gene	Target Name
IGSF9B	immunoglobulin superfamily member 9B
SPATS2L	spermatogenesis associated serine rich 2 like
CACNA1C	calcium voltage-gated channel subunit alpha1 C
PCNX1	pecanex 1
TMTC1	transmembrane O-mannosyltransferase targeting cadherins 1
DCC	DCC netrin 1 receptor
CPEB1	cytoplasmic polyadenylation element binding protein 1
NDST3	N-deacetylase and N-sulfotransferase 3
CNNM2	cyclin and CBS domain divalent metal cation transport mediator 2
AS3MT	arsenite methyltransferase
NMB	neuromedin B
TYW5	tRNA-yW synthesizing protein 5
CCDC122	coiled-coil domain containing 122
SF3B1	splicing factor 3b subunit 1
CENPM	centromere protein M
ARL3	ADP ribosylation factor like GTPase 3
PCDHA1	protocadherin alpha 1
ACTR5	actin related protein 5
GIGYF1	GRB10 interacting GYF protein 1
NAGA	alpha-N-acetylgalactosaminidase
GLYCTK	glycerate kinase
ALPK3	alpha kinase 3
DDHD2	DDHD domain containing 2
NEK4	NIMA related kinase 4
PCCB	propionyl-CoA carboxylase subunit beta
PRR12	proline rich 12
BAG5	BAG cochaperone 5
ASF1A	anti-silencing function 1A histone chaperone
PCDHA2	protocadherin alpha 2
PLPP5	phospholipid phosphatase 5
GNL3	G protein nucleolar 3
MST1R	macrophage stimulating 1 receptor
NAT8	N-acetyltransferase 8 (putative)
PSMA4	proteasome 20S subunit alpha 4
PITPNM2	phosphatidylinositol transfer protein membrane associated 2
PCDHA8	protocadherin alpha 8
XRCC3	X-ray repair cross complementing 3
SMDT1	single-pass membrane protein with aspartate rich tail 1
AP3B2	adaptor related protein complex 3 subunit beta 2
FAM221A	family with sequence similarity 221 member A
MAPK3	mitogen-activated protein kinase 3
KTN1	kinectin 1
PPM1M	protein phosphatase, Mg2+/Mn2+ dependent 1M
YJEFN3	YjeF N-terminal domain containing 3
TM6SF2	transmembrane 6 superfamily member 2
LRRC37A	leucine rich repeat containing 37A
NDUFAF7	NADH:ubiquinone oxidoreductase complex assembly factor 7
FOXN2	forkhead box N2
ELFN1	extracellular leucine rich repeat and fibronectin type III domain containing 1
CHRNA2	cholinergic receptor nicotinic alpha 2 subunit
ACE	angiotensin I converting enzyme
DNPH1	2'-deoxynucleoside 5'-phosphate N-hydrolase 1
IDH3B	isocitrate dehydrogenase (NAD(+)) 3 non-catalytic subunit beta
CDIP1	cell death inducing p53 target 1
KANSL1	KAT8 regulatory NSL complex subunit 1
INO80E	INO80 complex subunit E
CEBPZOS	CEBPZ opposite strand
TBX1	T-box transcription factor 1
LRRC37A2	leucine rich repeat containing 37 member A2
PSMG1	proteasome assembly chaperone 1
POLG	DNA polymerase gamma, catalytic subunit
TNFRSF13C	TNF receptor superfamily member 13C
GRM1	glutamate metabotropic receptor 1
PTK6	protein tyrosine kinase 6
MRM2	mitochondrial rRNA methyltransferase 2
WDR55	WD repeat domain 55
NPY	neuropeptide Y
GOLGA6L10	golgin A6 family like 10
NMRAL1	NmrA like redox sensor 1
REEP2	receptor accessory protein 2
RHOA	ras homolog family member A
PTK7	protein tyrosine kinase 7 (inactive)
CHST9	carbohydrate sulfotransferase 9
PPP3CC	protein phosphatase 3 catalytic subunit gamma
ZMAT2	zinc finger matrin-type 2
TMEM241	transmembrane protein 241
ZBED4	zinc finger BED-type containing 4
YPEL3	yippee like 3
XPNPEP3	X-prolyl aminopeptidase 3
RBM26	RNA binding motif protein 26
FAM83H	family with sequence similarity 83 member H
PPIL2	peptidylprolyl isomerase like 2
GCH1	GTP cyclohydrolase 1
GALNT1	polypeptide N-acetylgalactosaminyltransferase 1

Table S5: The 81 candidate gene lists for LDL-C validated by the Open Targets Validation Platform.

Gene	Target Name
ANGPTL3	angiopoietin like 3
RRBP1	ribosome binding protein 1
LPA	lipoprotein(a)
GIGYF1	GRB10 interacting GYF protein 1
DNAH11	dynein axonemal heavy chain 11
SLC22A3	solute carrier family 22 member 3
GAS6	growth arrest specific 6
INSIG2	insulin induced gene 2
LIPC	lipase C, hepatic type
L3MBTL3	L3MBTL histone methyl-lysine binding protein 3
ST3GAL4	ST3 beta-galactoside alpha-2,3-sialyltransferase 4
ABO	ABO, alpha 1-3-N-acetylgalactosaminyltransferase and alpha 1-3-galactosyltransferase
PPP1R3B	protein phosphatase 1 regulatory subunit 3B
NYNRIN	NYN domain and retroviral integrase containing
SP4	Sp4 transcription factor
PAQR9	progestin and adipoQ receptor family member 9
CELSR2	cadherin EGF LAG seven-pass G-type receptor 2
ZNF329	zinc finger protein 329
RHCE	Rh blood group CcEe antigens
RHD	Rh blood group D antigen
PLEC	plectin
EFCAB13	EF-hand calcium binding domain 13
PKN3	protein kinase N3
FADS1	fatty acid desaturase 1
SEC16A	SEC16 homolog A, endoplasmic reticulum export factor
DNAJC13	DnaJ heat shock protein family (Hsp40) member C13
PXK	PX domain containing serine/threonine kinase like
UBXN2B	UBX domain protein 2B
PSRC1	proline and serine rich coiled-coil 1
ST13	ST13 Hsp70 interacting protein
NF1	neurofibromin 1
DMTN	dematin actin binding protein
DPP3	dipeptidyl peptidase 3
PKD1L3	polycystin 1 like 3, transient receptor potential channel interacting
NECTIN2	nectin cell adhesion molecule 2
TNKS	tankyrase
CYSTM1	cysteine rich transmembrane module containing 1
ELOC	elongin C
CHD6	chromodomain helicase DNA binding protein 6
CLPTM1	CLPTM1 regulator of GABA type A receptor forward trafficking
RAB2A	RAB2A, member RAS oncogene family
PCMTD2	protein-L-isoaspartate (D-aspartate) O-methyltransferase domain containing 2
ABHD12	abhydrolase domain containing 12, lysophospholipase
PEX6	peroxisomal biogenesis factor 6
NT5DC1	5'-nucleotidase domain containing 1
CEACAM20	CEA cell adhesion molecule 20
CWF19L1	CWF19 like cell cycle control factor 1
PLTP	phospholipid transfer protein
SORT1	sortilin 1
GNMT	glycine N-methyltransferase
BMPR2	bone morphogenetic protein receptor type 2
PMPCA	peptidase, mitochondrial processing subunit alpha
PHLPP2	PH domain and leucine rich repeat protein phosphatase 2
CPNE3	copine 3
SRRT	serrate, RNA effector molecule
SCAMP5	secretory carrier membrane protein 5
MKRN2	makorin ring finger protein 2
PFDN1	prefoldin subunit 1
TMEM116	transmembrane protein 116
LPIN3	lipin 3
SLC2A4RG	SLC2A4 regulator
CBLN3	cerebellin 3 precursor
PSMG1	proteasome assembly chaperone 1
KPNB1	karyopherin subunit beta 1
SURF1	SURF1 cytochrome c oxidase assembly factor
KRI1	KRI1 homolog
NUDCD3	NudC domain containing 3
BLOC1S2	biogenesis of lysosomal organelles complex 1 subunit 2
SLC44A2	solute carrier family 44 member 2 (CTL2 blood group)
KLB	klotho beta
EMILIN3	elastin microfibril interfacer 3
STAT3	signal transducer and activator of transcription 3
PPCDC	phosphopantothenoylcysteine decarboxylase
MAPK3	mitogen-activated protein kinase 3
ZDHHC12	zinc finger DHHC-type palmitoyltransferase 12
CPNE1	copine 1
EXOC3L2	exocyst complex component 3 like 2
CEACAM19	CEA cell adhesion molecule 19
NPM2	nucleophosmin/nucleoplasmin 2
RPS6KL1	ribosomal protein S6 kinase like 1
TCTN3	tectonic family member 3

References

Candes, E., Fan, Y., Janson, L., and Lv, J. (2018). Panning for gold:'model-x'knockoffs for high dimensional controlled variable selection. *Journal of the Royal Statistical Society: Series B (Statistical Methodology)*, 80(3):551–577.
